# Factors associated with resilience among parents of children with autism spectrum disorder: A systematic review

**DOI:** 10.1371/journal.pone.0351969

**Published:** 2026-06-22

**Authors:** Nora Saliza Md Salim, Idayu Badilla Idris, Norazlin Kamal Nor, Syakirah Roslan, Nafisah Abdul Rashid, Roszita Ibrahim, Fairuz Nazri Abd Rahman

**Affiliations:** 1 Department of Public Health Medicine, Faculty of Medicine, Universiti Kebangsaan Malaysia, Kuala Lumpur, Malaysia; 2 Department of Paediatrics, Faculty of Medicine, Universiti Kebangsaan Malaysia, Kuala Lumpur, Malaysia; 3 Hospital Tunku Ampuan Besar Tuanku Aishah Rohani, Hospital Pakar Kanak-Kanak, Kuala Lumpur, Malaysia; 4 Department of Psychiatry, Faculty of Medicine, Universiti Kebangsaan Malaysia, Kuala Lumpur, Malaysia; IRCCS Medea: Istituto di Ricovero e Cura a Carattere Scientifico Eugenio Medea, ITALY

## Abstract

Parents of children with autism spectrum disorder (ASD) experience ongoing caregiving demands and challenges that may contribute to their general well-being. Research has shown that resilience enables parents to adapt to these challenges; however, existing evidence on associated factors toward resilience among these parents remains fragmented. This systematic review aimed to identify factors associated with resilience among parents of children with ASD. We conducted a PRISMA 2020-guided systematic review on studies published between January 2019 and May 2025 using Web of Science, Scopus, and PubMed by applying predefined search terms and Boolean operators. Studies examining factors associated with resilience among parents of children with ASD aged under 18 years were included in this review. We also used the Mixed Methods Appraisal Tool 2018 to assess the quality of these studies. Findings were synthesised narratively using thematic analysis. From the 13 selected studies, five themes were identified as determinants of resilience among parents, i.e., sociodemographic and economic factors; child-related characteristics; parenting well-being and distress; psychological factors and coping styles; and social support and family relationships. Most included studies were cross-sectional and utilised different measurement tools, which may limit causal inference and comparability. As a conclusion, parental resilience was found to be influenced by psychological, social, and contextual factors. Strengthening coping resources and social support may enhance parental adaptation and family well-being. Along these lines, multi-level and culturally sensitive interventions are thus needed to inform strategies to foster resilience among parents of children with ASD. The review was registered with PROSPERO (CRD420251034348). The author(s) received no specific funding for this work.

## Introduction

Autism spectrum disorder (ASD) is a complex neurodevelopmental condition characterised by a unique combination of impairments in social interactions and communication, including the presence of restricted and repetitive patterns of behaviour [1]. Globally, the prevalence of ASD is currently estimated at 1 in 127 persons [2], and the rising trend of ASD among children over the recent years is a growing public health concern [3–6]. Additionally, more parents are now involved in raising children with ASD. Although ASD is a lifelong condition parenting a child with ASD presents unique challenges that may significantly affect parents’ overall well-being and their quality of life [7]. Studies consistently showed that parents of children with ASD experienced higher levels of stress as compared to parents of typically developing children or those with other developmental disabilities [8–11]. Moreover, parents of children with ASD frequently encounter emotional consequences, including persistent stress, heightened anxiety, and strained interpersonal relationships, all of which can affect overall parenting and family functioning [12]. Caregiving demands, encompassing financial challenges and related stressors, were also shown to be significant factors contributing to reduced quality of life among parents of children with ASD [13–15].

Resilience, which is defined as the ability to recover from adverse events, threats, or challenges, is a significant protective factor that enables parents’ positive adaptation in facing the hurdles of caring for their children, and it is also found that resilience can promote favourable health outcomes [16]. However, the degree of resilience varies widely across individuals, suggesting that multiple factors may influence parents’ ability to adapt to stressors. Parents with high resilience were able to go through life challenges and cope effectively when faced with any difficulties [17]. Research has shown that higher resilience allows parents to maintain an optimistic mental outlook and overall emotional and general well-being [18] and consequently improve their quality of life [19]. Enhancing parental resilience can optimise the quality of care provided to children with ASD, despite challenging circumstances [20].

Despite increasing research on resilience, evidence on its contributing factors among parents of children with ASD remains fragmented. Previous existing literature focused on parenting stress rather than parental resilience [21]. Other literature examined resilience among parents of children with developmental disabilities in general, rather than specifically within the context of ASD [22], or assessed resilience within the family level instead of focusing exclusively on parents [23,24]. This review will focus on resilience among parents of children with ASD, who are generally the primary caregivers and thus experience the most direct and enduring caregiving impacts [25]. According to Parental Investment Theory [26], greater parental involvement in caregiving may strengthen emotional bonds, which in turn amplifies the effects of caregiving compared to that of other family members. Similarly, the Attachment Theory [27] emphasises parents as the principal attachment figures, who are most likely to establish secure relationships with their children. The inclusion of other caregivers, such as grandparents or siblings, who may have different caregiving responsibilities and levels of emotional involvement [28], could introduce contextual variability that falls beyond the scope of this review’s focus on parental experiences.

Considering this gap, this review aims to comprehensively identify, appraise, and synthesise existing literature on factors associated with parental resilience. These findings are expected to provide valuable insights for future programmes and interventions among parents of children with ASD with the ultimate goal of strengthening their resilience.

## Materials and methods

### Protocol and registration

This systematic literature review was conducted and reported in accordance with the Preferred Reporting Items for Systematic Reviews and Meta-Analyses (PRISMA) 2020 statement [29]. The PRISMA 2020 checklist is provided in S1 Checklist. The abstract was prepared according to the PRISMA 2020 for abstract reporting guideline (S2 Checklist). The review was registered in the International Prospective Register of Systematic Reviews (PROSPERO; CRD420251034348). No separate protocol was published. Minor administrative amendments were made to the registry to reflect methodological clarifications.

### Eligibility criteria

Studies were eligible if they met the following criteria: (1) participants included only parents (father, mother, or both) of children diagnosed with ASD; (2) the children with ASD were younger than 18 years of age; (3) the studies examined factors associated with parental resilience; and (4) resilience was reported as an outcome. Quantitative, qualitative, and mixed-methods studies which were published in peer-reviewed journals between 1 January 2019 and 2 May 2025 were included. No language restrictions were applied. Studies were excluded if they: (1) involved parents of children with developmental disabilities other than ASD; (2) included caregivers other than parents; (3) did not report resilience as an outcome; or (4) were non-original research.

### Search strategy

The search strategy was developed using the Population, Exposure, and Outcome (PEO) framework, which provided a structured approach for identifying key concepts relevant to this review (Table 1). The three concepts included parents of children with ASD, factors associated with resilience, and resilience as the outcome. Comprehensive keywords were generated for each concept, and truncation symbols (*) were used to capture variations of the terms. The Boolean operators (AND, OR) were applied across databases and the syntax was adapted according to each database’s specific requirement.

**Table 1:** Population, exposure, and outcome framework.

Elements	Description	Keywords
Population	Parents of children with ASD	parent*, mother*, father*, autism spectrum disorder*, autistic, autism, autistic disorder, neurodevelopmental disorder*, asperger's, high-functioning autism
Exposure	Factors associated with resilience	factor*, associate*, related factor*, contributing factor*, determinant*, influencing factor*, predictor*, contributor*, risk factor*, protective factor*
Outcome	Resilience	resilien*, adaptability, adaptation, perseverance, toughness, emotional strength, hardiness, psychological resilience, emotional resilience, psychological adaptation, psychological adjustment, stress resistance, psychological strength

This review utilised multiple electronic databases, i.e., Web of Science, Scopus, PubMed, and selected databases which were accessed via the EBSCOhost platform, which include MEDLINE Complete, Psychology and Behavioral Sciences Collection, SocINDEX with Full Text, Education Research Complete, and Academic Search Complete.

The final search was conducted on 2 May 2025. The full search strategy for each database is provided in S1 Table.

### Study selection

All studies that were identified through database searches were managed in Microsoft Excel, where duplicates were removed. Two reviewers independently screened titles and abstracts based on the predefined criteria, followed by a full-text review of eligible articles by other reviewers. Any disagreements between reviewers were resolved through discussions until a final consensus was reached.

### Data extraction

Data from the included studies were systematically extracted into a structured Microsoft Excel sheet. Two reviewers independently performed data extraction, and discrepancies were resolved through discussion to ensure accuracy. The extracted information included study characteristics (author(s), year of publication, country, study objective and design, sample size), population characteristics (age, gender, relationship to the child, ASD severity), exposure variables (factors associated with resilience), the primary outcome of interest (resilience) and the key findings. All data were recorded as reported in the original studies.

### Data synthesis

Findings were tabulated to organized the data and facilitate comparison across studies. Statistical results from quantitative studies, including odds ratios, regression coefficients, and correlation values, were recorded. For qualitative studies, the participant narratives and thematic categories, were extracted. No data conversion was performed. The findings were coded and organised into general themes that represented factors associated with parental resilience. This approach facilitated the triangulation of findings and identification of similar, complementary, or even contrasting evidence across study designs.

### Article quality appraisal

The Mixed Methods Appraisal Tool (MMAT, 2018) was used to evaluate the study quality [30]. This tool was selected as the review included diverse study designs, enabling consistent and comprehensive appraisal across qualitative, quantitative, and mixed-methods studies. The MMAT consists of five criteria for each study design, rated as “Yes”, “No” or “Can’t tell”. Two reviewers independently appraised each study. Any discrepancies were resolved through discussions or consultation with a third reviewer.

## Results

**Fig 1:**
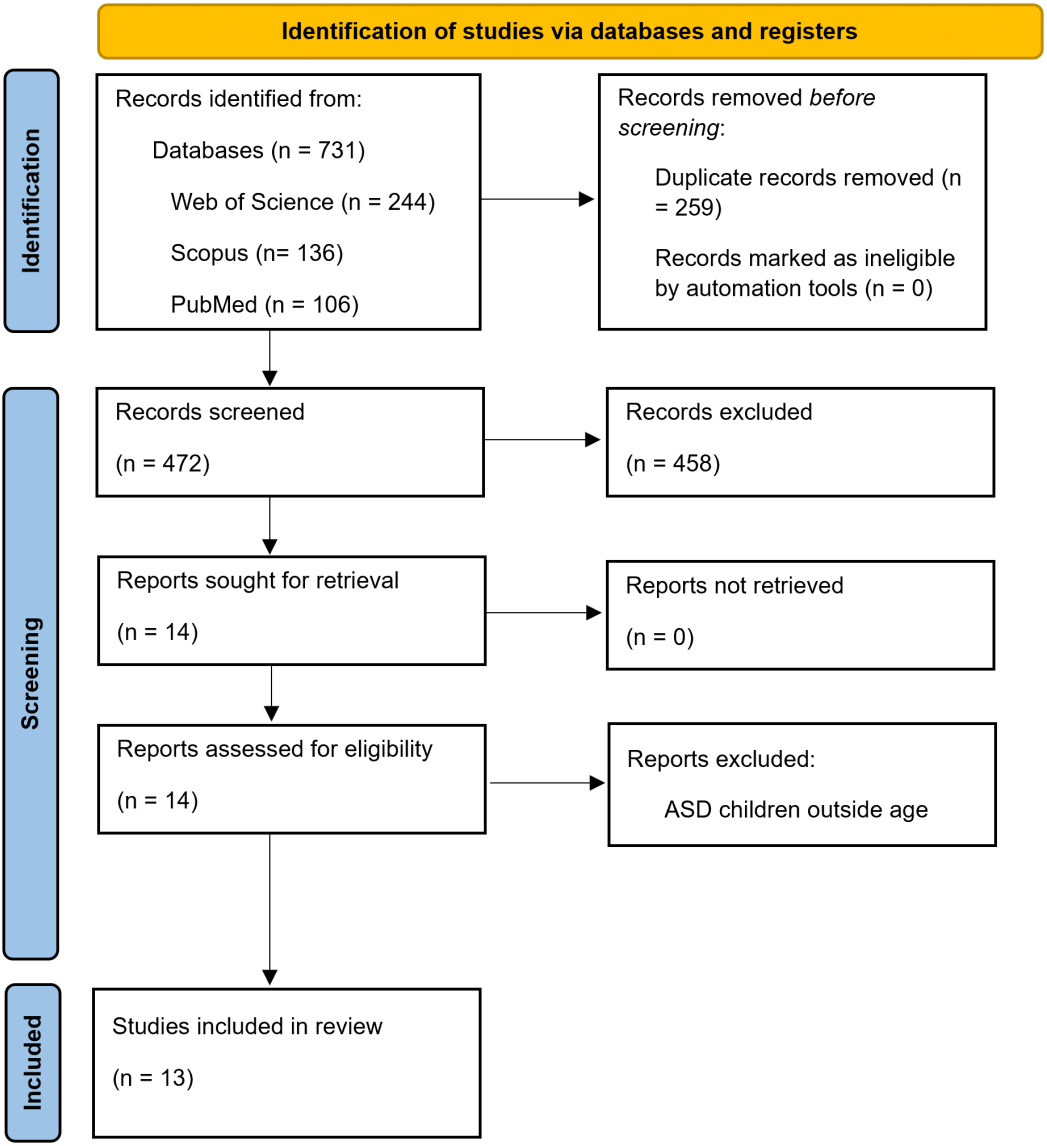
PRISMA 2020 flow diagram of the article selection process.

A total of 731 records were identified through electronic database searches. After removing duplicates records (n = 259), 472 records remained for title and abstract screening. Of these, 458 records were excluded based on the predefined inclusion criteria. Fourteen reports were sought for full-text assessment, and all were retrieved. Following full-text assessment for eligibility, one study was excluded because it involved children with ASD who were older than 18 years of age. Ultimately, 13 studies met all inclusion criteria and were included in this review (Fig 1).

### Study characteristics

The included studies were conducted across eight countries, i.e., China (n = 4) and Spain (n = 3), with one study each from Canada, Croatia, India, Iran, Malaysia, and the United States. Out of all these studies, 11 employed quantitative designs and two used qualitative approaches. Although some articles were part of a broader mixed-methods projects, all eligible publications reported either quantitative or qualitative findings exclusively.

Sample sizes ranged from 5 to 799 participants, with the majority involving mothers of children with ASD. Quantitative studies utilised various validated instruments to measure resilience and related factors, using measurement tools based on the study objectives (Table 2). In contrast, qualitative studies explored parents’ lived experiences and other influencing factors without using standardised measures.

**Table 2:** Characteristics of included studies.

Author (Year)	Country	Study design	Sample size	Parent characteristics	Child characteristics	Tool(s) used to measure resilience	Tool(s) used to measure factors
Aithal et al. (2020) [31]	India	Qualitative	6	6 mothers Mean age 29.6 years, SD not reported (range 28–35)	Gender distribution not reported Mean age 7.2 years (SD and range not reported) Severity not reported	Not applicable	Interview – collected qualitative data on parental experiences.
Pastor-Cerezuela et al. (2020) [32]	Spain	Quantitative (cross-sectional)	97 ASD: 32 DS: 23 TD: 42	ASD: 27 mothers, 5 fathers Mean age 39.09 ± 4.29 years (range not reported) DS: 19 mothers, 4 fathers Mean age 46.17 ± 4.28 years (range not reported) TD: 33 mothers, 9 fathers Mean age 39.98 ± 4.89 years (range not reported)	ASD: 29 males, 3 females Mean age 6.79 ± 1.08 years (range not reported) Severity: mild to moderate DS: 15 males, 8 females Mean age 8.52 ± 2.54 years (range not reported) TD: 34 males, 8 females Mean age 6.82 ± 1.16 years (range not reported)	Resilience Scale	Parental Stress Index – measured parenting stress level.
Flores-Buils and Andres-Roqueta (2022) [33]	Spain	Quantitative (case-control)	156 NDD: 73 (ASD 36, ADHD 20, DLD 17) TD (control group): 73	Not mentioned	NDD: 57 males and 16 females ASD: mean age 106.78 ± 25.41 months (range 60–154). Severity not reported. ADHD: mean age 117.05 ± 22.88 months (range 74–155). DLD: Mean age 99.12 ± 31.85 months (range 60–153) TD: Gender distribution not reported Mean age 110.45 ± 24.42 months (range 63–146)	Connor-Davidson Resilience Scale-25	Contextual data form (study-specific) – assessed formal and informal support, and number of children. Test for Reception of Grammar – measured structural language. Test of Emotion Comprehension – measured social cognition.
Ji et al. (2022) [34]	China	Quantitative (cross-sectional)	182	145 mothers, 37 fathers Mean age 32.16 years (SD and range not reported) for parents with child age < 6 years Mean age 37.83 years (SD and range not reported) for parents with child age > 6 years	140 males, 42 females Mean age 5.37 years (SD and range not reported) with 64.29% were less than six years old Severity (perceived by parents): 49 autistic features, 28 mild, 54 moderate, 21 severe, 30 others	Connor-Davidson Resilience Scale-25	Sociodemographic questionnaire – collected parental data (age, gender, parent-child relationship, marital status, educational level, employment status, place of residence, number of children, family income, and caring time) and child’s data (age, gender, and level of autism diagnosis). Affiliate Stigma Scale – assessed perceived social stigma.
Schwartzman et al. (2022) [35]	United States	Quantitative (cross-sectional study, secondary analysis of RCT baseline data)	50	35 mothers, 15 fathers Mean age 40.6 ± 3.6 years (age range 35–56 years)	42 males, 8 females Mean age 6.9 ± 1.4 years (range not reported) Severity not reported	Connor-Davidson Resilience Scale-25	Psychological functioning Depression, Anxiety, Stress Scale-21 – measured severity of depression, anxiety, and stress symptoms in the past two weeks. Broad Autism Phenotype Questionnaire – assessed autism traits in parents. Child functioning and parenting stress Social Responsiveness Scale-2 – assessed child social impairment. Aberrant Behaviour Checklist-2 – measured maladaptive behaviour. Parenting Stress Index, Fourth Edition, Short Form – measured parenting stress level. Positive mental health practices Action and Acceptance Questionnaire, Second Edition – assessed psychological flexibility and acceptance. Mindfulness Attention Awareness Scale – measured present moment awareness and mindfulness in daily life. Life Orientation Test-Revised – measured optimism. Self-Compassion Scale – Short Form – measured self-compassion.
Ghanouni and Eves (2023) [36]	Canada	Quantitative (cross-sectional)	50	49 mothers, 1 father Mean age 38.66 ± 7.59 years (range 26–65)	42 males, 8 females Mean age 7.52 ± 2.71 years (range 2–11). Severity not reported.	Parenting Resilience Elements Questionnaire	Demographic questions (age, sex, ethnicity, number of children with disabilities at home, monthly household income, their children’s age, sex, and children’s background). Parental Stress Scale – measured parenting stress level. Strengths and Difficulties Questionnaire – measured children’s difficulty level as an index of severity. Child and Youth Resilience Measure – measured of resilience indicators in children as reported by their primary caregiver. Child and Adolescent Scale of Participation – measured child’s participation in different contexts.
Rasoulpoor et al. (2023) [37]	Iran	Quantitative (cross-sectional)	69	69 mothers Mean age 38.4 ± 7 years (range 26–54 years)	55 males, 14 females Age: 3-6 years old: 21 7-10 years old 17 11-15 years old: 31 Mean age and SD not reported Severity not reported	Connor-Davidson Resilience Scale-25	Caregiver burden inventory – measured objective and subjective burden of care.
Žic Ralić and Matišić (2023) [38]	Croatia	Quantitative (cross-sectional)	70	65 mothers, 5 fathers Age range 25–55 years (mean and SD not reported)	Gender not reported Age range 3–17 years old (mean age and SD not reported) Severity (based on parental report of support needs in daily functioning): 48.6% required full support, 24.3% high support, 22.9% moderate support, and 4.3% little support.	Family Resilience Assessment Scale	General Questionnaire on characteristics of a child with autism – measured child functioning (severity of symptoms across communication, socialization, need for rigid routines, and frequency of problem behaviours). Multidimensional Stress Questionnaire for Couples – measured general stress across eight domains (work/education, social contacts, leisure, children, life situations, environment, income, daily struggles). Parental Stress Scale
Lam et al. (2024) [39]	China	Qualitative	16	11 mothers, 5 fathers Mean age 42.36 ± 7.02 years (range 29–53)	12 males, 1 female Mean age 8.08 ± 3.71 years (range 3–13) Severity not reported	N/A	Interview
Salleh et al. (2024) [19]	Malaysia	Quantitative (cross-sectional)	144	111 mothers, 33 fathers Mean age 36.4 ± 5.78 years (range 23–54)	119 males, 25 females Mean age 6.17 ± 2.75 years (range not reported) Severity perceived by parents (scale 1–10): mean 4.83 ± 1.82	Connor-Davidson Resilience Scale-25	Affiliate Stigma Scale Quality of Life in Autism-Parent Version – measured parental quality of life related to raising a child with autism.
Chen et al. (2025) [40]	China	Quantitative (cross-sectional)	254	195 mothers, 59 fathers Mean age 35.74 ± 7.20 years (range 19–70)	203 males, 51 females Mean age 5.63 ± 2.73 years (range 3–14 years) Severity not reported	Family resilience assessment scale	Zarit Burden Interview – assessed the burden experienced by caregivers. Social support rate scale – measured social support across subjective (perceived support), objective (networks and material aid), and utilization (seeking and using support) dimension. Illness Cognition Questionnaire-Parent version – measured parental illness cognition across helplessness, acceptance, and perceived benefits subscales.
Liu et al. (2025) [41]	China	Quantitative (cross-sectional)	229	176 mothers, 53 fathers Mean age (mother) 34.84 ± 5.40 (range not reported) Mean age (father) 37.45 ± 6.09 (range not reported)	171 males and 58 females Mean age 5.94 ± 3.09 years (range not reported) Severity: 183 mild to moderate, 46 severe	Family resilience assessment scale	Sociodemographic questionnaire – collected parental data (age, education, employment, religion, monthly income, number of children, family type) and child data (age, gender, age of diagnosis, severity of illness, treatment cost).
Sarhani-Robles et al. (2025) [42]	Spain	Quantitative (cross-sectional)	799	421 mothers, 378 fathers Mean age 36.79 ± 11.81 years (range 26–70)	Gender, age and severity not reported	Resilience Scale (RS-14)	Sociodemographic questionnaire – collected information on gender, age, educational level, employment status, presence of COVID-19 infection, and type of housing. Psychological Well-being Scale – measured satisfaction with life. Positive Mental Health Scale – evaluated emotional, psychological, and social well-being. Coping Humour Scale – assessed the use of humour in coping with stressful situations. Reappraisal Index – measured positive cognitive reappraisal across learning, effort, and reframing dimensions. Rosenberg Self-Esteem Scale – measured personal feelings of worth and self-respect. Lubben Social Network Scale – assessed perceived social support from friends and family members.

**Note.** SD = Standard deviation; ASD = autism spectrum disorder; DS = Down syndrome; TD = typical development; ADHD = attention deficit hyperactivity disorder; DLD = Developmental language disorder; NDD = Neurodevelopmental disorders

### Quality appraisal

The quality of all 13 included studies was assessed using the MMAT, version 2018. Overall, the studies demonstrated moderate to high quality, with most fulfilling four or more appraisal criteria (Table 3).

**Table 3:** Results of the quality assessment.

Study	Study design	QA1	QA2	QA3	QA4	QA5	Number of criteria fulfilled
Aithal et al. [31]	QL	**Y**	**Y**	**Y**	**Y**	**Y**	**5/5**
Pastor-Cerezuela et al. [32]	QN (NR)	**Y**	**Y**	**Y**	**Y**	**Y**	**5/5**
Flores-Buils and Andres-Roqueta (33)	QN (NR)	**Y**	**Y**	**Y**	**Y**	**Y**	**5/5**
Ji et al. [34]	QN (NR)	**C**	**Y**	**Y**	**Y**	**Y**	**4.5/5**
Schwartzman et al. [35]	QN (NR)	**C**	**Y**	**Y**	**Y**	**Y**	**4.5/5**
Ghanouni and Eves (36)	QN (NR)	**C**	**Y**	**Y**	**C**	**Y**	**3.5/5**
Rasoulpoor et al. [37]	QN (NR)	**C**	**Y**	**Y**	**N**	**Y**	**3.5/5**
Žic Ralić and Matišić (38)	QN (NR)	**C**	**Y**	**Y**	**N**	**Y**	**3.5/5**
Lam et al. [39]	QL	**Y**	**Y**	**Y**	**Y**	**Y**	**5/5**
Salleh et al. [19]	QN (NR)	**Y**	**Y**	**Y**	**Y**	**Y**	**5/5**
Chen et al. [40]	QN (NR)	**C**	**Y**	**Y**	**Y**	**Y**	**4.5/5**
Liu et al. [41]	QN (NR)	**C**	**Y**	**Y**	**Y**	**Y**	**4.5/5**
Sarhani-Robles et al. [42]	QN (NR)	**C**	**Y**	**Y**	**Y**	**Y**	**4.5/5**

**Note.** QA = quality assessment criterion; QL = qualitative; QN (NR) = quantitative non-randomised; Y = yes; N = no; C = can’t tell.

### The developed themes

The extracted study-level findings from the included studies are provided in S2 Table. Thematic synthesis revealed five overarching themes that influenced parental resilience which were (1) sociodemographic and economic factors; (2) child-related characteristics; (3) parenting well-being and distress; (4) psychological factors and coping styles; and (5) social support and family relationships (Table 4). Each theme encompasses factors associated with varying levels of parental resilience (Table 5).

**Table 4:** Themes of factors associated with parental resilience in ASD.

Author (Year)	Theme 1: Socio-demographic and economic factors	Theme 2: Child-related characteristics	Theme 3: Parenting well-being and distress	Theme 4: Psychological factors and coping styles	Theme 5: Social support and family relationships
Aithal et al. (2020) [31]				**✔**	
Pastor-Cerezuela et al. (2020) [32]			**✔**		
Flores-Buils and Andres-Roqueta (2022) [33]					**✔**
Ji et al. (2022) [34]			**✔**		
Schwartzman et al. (2022) [35]			**✔**	**✔**	
Ghanouni and Eves (2023) [36]	**✔**		**✔**		
Rasoulpoor et al. (2023) [37]			**✔**		
Žic Ralić and Matišić (2023) [38]		**✔**			
Lam et al. (2024) [39]	**✔**		**✔**	**✔**	**✔**
Salleh et al. (2024) [19]		**✔**	**✔**		
Chen et al. (2025) [40]			**✔**	**✔**	**✔**
Liu et al. (2025) [41]	**✔**	**✔**			
Sarhani-Robles et al. (2025) [42]	**✔**			**✔**	**✔**

**Table 5:** Thematic synthesis of the reviewed studies.

Theme	Subtheme	Key findings	Authors
Theme 1: Socio-demographic and economic factors	Age	Parents aged 48–58 years had significantly higher resilience levels compared to younger parents (β = 3.03, p < 0.01).	Sarhani-Robles et al. [42]
	Employment status	Self-employed was positively associated with resilience (β = 1.17, p < 0.01). Father’s employment reduced the odds of being in the low-resilience group (OR = 0.134, p = 0.035), indicating a protective effect on family resilience. Unemployment increased financial stress burden among parents.	Lam et al. [39]; Liu et al. [41]; Sarhani-Robles et al. [42]
	Financial burden	High treatment costs were associated with greater odds of low resilience (OR = 6.915, p = 0.046) and moderate resilience (OR = 5.750, p = 0.032), compared with high resilience. High cost of treatment and education contributed to substantial economic pressure on parents.	Lam et al. [39], Liu et al. [41]
	Income	Higher household income was positively associated with resilience (β = 0.37, p = 0.01). Single-income households experienced greater financial strain and heightened stress. COVID-19-related wage reductions exacerbated financial burden.	Ghanouni and Eves (36); Lam et al. [39]
Theme 2: Child-related characteristics	Severity	Mild-moderate illness severity was associated with higher odds of resilience (OR = 0.227, p = 0.009). High severity of ASD was associated with lower resilience (β = −2.353, p < 0.001).	Salleh et al. [19]; Liu et al. [41]
	Comorbidities	Presence of comorbidities was associated with lower resilience (β = -7.375, p = 0.030).	Salleh et al. [19]
	Disability card status	Having a disability card was associated with lower resilience (β = −4.558, p = 0.038).	Salleh et al. [19]
	Behavioural changes	Problem behaviours were negatively correlated with resilience, affecting family communication and problem-solving (r = −0.289, p < 0.05), ability to find meaning in adversity (r = −0.289, p < 0.05), and family connectedness (r = −0.252, p < 0.05). Independence from rigid routines was positively correlated with resilience, improving family communication and problem-solving (r = 0.249, p < 0.05), meaning-making (r = 0.362, p < 0.05), and connectedness (r = 0.352, p < 0.05). Greater social interest was positively correlated with resilience (r = 0.355, p < 0.01), supporting family connectedness.	Žic Ralić and Matišić (38)
Theme 3: Parenting well-being and distress	Parenting stress	Higher parenting stress was negatively associated with resilience (β = −0.40, p < 0.01; β = −0.80, p < 0.001; r = −0.381, p = 0.031). Parents reported high stress due to lack of ASD knowledge among parents, trouble dealing with their child’s behaviour, and the demands of being the primary caregiver.	Pastor-Cerezuela et al. [32]; Schwartzman et al. [35]; Ghanouni and Eves (36); Lam et al. [39]
	Parenting Burden	Higher caregiver burden was negatively associated with resilience (β = −0.417, p < 0.001; r = −0.536, p < 0.001).	Rasoulpoor et al. [37]; Chen et al. [40]
	Stigma	Affiliate stigma was negatively correlated with resilience (r = −0.51 to −0.469, p < 0.001). 37.5% of parents reported experiencing social stigma, including negative labelling by the community, peer rejection, and unpleasant public encounters.	Ji et al. [34]; Lam et al. [39]; Salleh et al. [19]
Theme 4: Psychological factors and coping styles	Psychological resources	Emotion regulation, sense of humour, optimism, self-compassion, and positive mental health were significantly associated with higher resilience (β = 8.36, 6.11, 0.37, 0.34, and 9.13; all p < 0.01). Positive cognition was positively associated with resilience (β = 0.179, p = 0.002). 62.5% of parents were aware of autism symptoms and sought diagnosis. Positive personality traits, including optimism, independence, an easy-going nature, and patience, promoted tolerance and acceptance of imperfection throughout the caregiving experience.	Schwartzman et al. [35]; Lam et al. [39]; Chen et al. [40]; Sarhani-Robles et al. [42]
	Psychological barriers	Anxiety was negatively associated with resilience (β = −0.61, p < 0.01). Parents expressed worry about their child’s future, including their independence, employment and long-term care needs, which contributed to distress and could negatively affect resilience. 68.75% of parents experienced anxiety, depression, grief, and hopelessness following a child’s diagnosis; 37.5% had physical symptoms such as sleep problems, nausea, and headache, which then hindered coping. Some parents ignored early autism symptoms despite being aware of them, treating their child as typically developing and delaying help-seeking and intervention.	Schwartzman et al. [35]; Lam et al. [39]
	Coping and adaptation	Dance movement therapy improved resilience by enhancing emotional release, self-awareness, and the development of positive coping strategies. 75% of parents used appraisal-focused coping by reframing their child’s condition positively, such as seeing concentration problems as normal for age. 93.75% used emotion-focused coping, including releasing emotions, mindfulness, meditation, and stress-relief activities like painting and exercising. 25% enrolled in courses and read autism-related books to learn caregiving strategies, although 12.5% reported that such courses were not always effective, as they were mismatched to their child’s needs. Four out of 16 parents use maladaptive coping, such as avoidance, giving up on tutoring tasks, and relying on medication to manage anxiety symptoms.	Aithal et al. [31]; Lam et al. [39]
Theme 5: Social support and family relationship	Formal support	Formal social support was positively associated with resilience (β = 0.42, p = 0.013). 37.5% of parents reported government autism rehabilitation courses were insufficient and required long waiting times, leads to limited effect. Parents sought help from social workers, autism-related associations, and welfare agencies. Proactive parents searched online for resources, while introverted parents were less likely to seek help.	Flores-Buils and Andres-Roqueta (33); Lam et al. [39]
	Informal support	Social support was positively associated with resilience (β = 9.28, p < 0.01; β = 0.181, p = 0.033). 100% of parents reported receiving some form of support from community, family, spouse, or friends. 75% of parents highlighted family support as important, by giving help in terms of caregiving, financial assistance, and unconditional acceptance of the child. 25% of parents reported no family support and chose not to disclose their child’s diagnosis to their parents. Some mothers reported making friends through community programs and autism support groups (e.g., WeChat groups, regular social gatherings, yoga, baking classes) which provided emotional and practical support.	Lam et al. [39]; Chen et al. [40]; Sarhani-Robles et al. [42]
	Marital relationship	Three parents described “empty love” relationships characterized by commitment without intimacy or passion. Husbands contributed financially but seldom engaged in caregiving. Seven parents reported marital conflict due to parenting disagreements or denial of the child’s condition, with some conflicts led to relationship deterioration or separation. 68.75% of parents reported supportive marital relationships where caregiving responsibilities were shared and worked together, strengthening family cohesion.	Lam et al. [39]

Taken together, these findings highlighted the differing factors influencing resilience among parents of children with ASD. Sociodemographic and economic factors such as advanced parental age, stable employment, and higher household income were associated with increased resilience, while unemployment and financial burden impaired coping capacity [36,39,41,42]. Child-related factors exhibited diverse correlations with parental resilience. Children with milder autistic symptoms and stronger social adjustment were associated with higher parental resilience, whereas the presence of comorbidities and challenging behaviour was linked to lower resilience [19,38,41]. Furthermore, stress, which was commonly associated with limited understanding of ASD, difficulties managing the child’s behaviour, the intensive caregiver demands, and perceived stigma, demonstrated a clear negative association with resilience [19,32,34–37,39,40]. In contrast, psychological factors such as emotion regulation, optimism, humour, self-compassion, positive mental health, and constructive cognition strengthened resilience, whereas anxiety, depression, and maladaptive coping hindered resilience [31,35,39,40,42]. Finally, strong social and marital support, including assistance from family, peers, and formal services, was identified as a key role in enhancing parental resilience [33,39].

## Discussion

This review synthesises evidence from 13 studies investigating factors associated with resilience amongst parents of children with ASD, indicating that resilience is influenced by a combination of individual and contextual factors, rather than acting as a single determinant. These findings are consistent with the Resiliency Model of Family Stress, Adjustment, and Adaptation, which emphasises the dynamic interplay between personal and environmental resources in shaping adaptation [43], and the Double ABCX Model of Family Stress and Adaptation [44], which suggests that adaptation relies on the optimal balance between risk factors, protective factors, and available resources.

Of the five themes identified, parenting well-being and distress was the most common theme, being reported in the greatest number of studies as a key factor influencing parental resilience. Parenting stress, caregiving burden, and stigma consistently acted as barriers to parents’ adaptive functioning, reducing their capacity to cope effectively with the demands of raising a child with ASD. Parents of children with disabilities commonly experienced psychological distress [45]. Consistently, findings from previous reviews showed higher stress levels amongst parents of children with ASD compared to parents of typically developing or other disabled children [46,47]. Parental mental health vulnerability may meaningfully influence resilience by increasing psychological strain and reducing parent’s capacity to perceive caregiving challenges positively, and use coping resources [48]. This aligns with stress-coping perspectives, which conceptualise caregiving as a continuous stress experience determined by individual vulnerabilities, available resources, and the intensity of caregiving demands [49]. Exposure to chronic and acute stressors depletes the psychological and physiological resources necessary for positive adaptation, thereby impairing emotional regulation, increasing perceptions of threat, and reducing persistence after various setbacks [50]. These findings are consistent with studies on caregivers of other chronic conditions, showing moderate association between caregiver burden and resilience [51–53]. Stigma, or a negative perception or sense of disapproval that a society places on a group or individual based on certain characteristics, which can be expressed through embarrassment, shame, and discrimination, may exacerbate parents’ psychological strain, leading to poorer health outcomes, reduced competence and belonging, undermining caregivers’ psychological adaptation across diverse contexts, and lower overall quality of life [54–57]. Similarly, evidence from other caregiving populations, including those caring for individuals with special needs [58] and developmental disabilities [59], also highlighted stigma as a salient predictor of reduced resilience.

Psychological resources are major facilitators of resilience, consistent with positive psychology literature, which identifies these traits as protective against stress [60,61]. From a broader theoretical perspective, Human Birth Theory suggests that, in the absence of severe early challenges, human beings may have original psychological resources that support natural resilience [62]. In ASD caregiving, this perspective may help explain why some parents respond to their child’s diagnosis with determination, actively seeking help, and have parenting commitment to improve child’s condition. Similar associations have been observed in other caregiving contexts, where psychological resources, including positive cognitions, optimism, and self-efficacy, enhance resilience [63]. Conversely, psychological distress, including anxiety, depressive symptoms, and emotional exhaustion, serves as a major barrier to resilience by depleting coping resources and diminishing adaptive capacity, which was aligned with findings among caregivers of chronic neurological patients [64]. Consistent with previous findings, problem-focused and cognitive coping strategies, such as acceptance and positive reappraisal, improve psychological adjustment and resilience, whereas emotion-focused coping strategies, including avoidance and denial, were linked to poorer mental health outcomes [65]. Regarding therapeutic interventions aimed at enhancing resilience, similar outcomes have been reported in a previous study using mobile-based psychological programs, which had effectively improved emotional regulation, self-awareness, and positive thinking [66].

Beyond individual factors, social support and family relationships emerged as important determinants of parental resilience. Formal and informal support networks consistently played vital roles, as mentioned by many studies, supporting the Buffering Hypothesis, which posits that social support protects psychological well-being by mitigating impacts of stress [67]. Previous evidence also confirms that social support is a critical protective factor, enhancing adaptive capacity under stress and promoting resilience among caregivers of children with developmental disabilities [22,68]. Similar findings were also observed in other caregiving populations, where stronger social support predicted greater resilience [69,70]. Furthermore, the quality of spousal and partner relationships emerged as another important protective factor, showing that supportive marriages enhanced parental resilience [71,72].

This review also found that sociodemographic and economic factors can significantly influence parental resilience. Similar findings in other caregiving contexts indicated that advanced age has been associated with higher resilience levels, suggesting that life experience enhances coping ability [73,74]. Additionally, employment stability provides financial security and work flexibility that helped alleviate caregiving stress [75]. A scoping review also found that higher income, better education, and sustained employment, facilitate resilience among informal caregivers [76]. On the other hand, high financial burden contributed by treatment fees, education, and caregiving, along with income loss, undermines parental resilience by increasing stress and limiting coping resources. Consistent findings indicate that financial strain lowers resilience, whereas economic stability promotes better psychological adjustment [21,64].

Another crucial factor that contributes to parental resilience is the child’s characteristics. Children often exhibit difficulties in communication, rigid interests, and limited self-awareness [77]. Parents of children exhibiting mild to moderate autistic symptoms reported higher resilience than those who have children with severe impairments. A review similarly identified child symptomatology as one of the strongest influences on caregiver well-being [23]. These findings suggest that greater symptom severity intensifies caregiving demands and emotional strain, which ultimately diminishes parents’ adaptive ability [21]. Recent evidence highlights that children’s adaptability and repetitive behaviours significantly predict caregiving burden and poorer psychological outcomes, thereby reinforcing the strong association between child functioning and parental resilience [78].

### Implications for practice and policy

The findings highlight important implications for clinical settings, community-based initiatives and the development of evidence-informed policies. Early ASD rehabilitation often requires parental involvement, including engagement with healthcare professionals, taking part in intervention planning, and consistent application of home-based or parent-mediated strategies [79,80]. Parent-mediated interventions are particularly important as they place parents in an active role in supporting their child’s social communication and developmental skills, while also enhancing parental competence, empowerment, and integration of strategies into daily routines [81]. Strengthening parental resilience may help parents to maintain their involvement despite emotional, practical, and financial challenges, thereby improving service uptake, adherence to rehabilitation recommendations, and continuity of intervention [82]. Strategies that enhance resilience among parents, such as routine screening for caregiver mental health status, referral to peer support groups, and psychoeducation, should be integrated into existing ASD care pathways. Clinicians should also be aware of the emotional and social challenges experienced by parent of children with ASD, and treat them with kindness, respect, and acknowledging their caregiving efforts. This approach may help parents feel supported rather than judged, and also strengthen trust, communication, and adherence with care pathway for their children [83,84]. Family therapy may also be considered as another support option for parents of children with ASD, particularly to enhance family communication, relationships, and coping when caregiving stress affects family functioning [85]. Collaboration between healthcare professionals and community organisations can improve access to peer networks and support resources, alleviate caregiver stress and enhance coping mechanisms. Policymakers should prioritise financial assistance, employment policies, and enhanced accessibility to services, therapy, and educational resources for children with ASD. All of these can be accomplished by expanding insurance coverage to include mental health services for caregivers, implementing paid caregiver leave to ensure financial stability and alleviate stress, and allocating federal or local funding to community support initiatives that promote resilience-building activities among families affected by ASD.

### Strengths and limitations

The significant strength of this review is the exclusive focus on parents only, enabling a more targeted synthesis of factors that are directly related to their resilience. This review provides an understanding of the integration between psychological, social, and contextual determinants that shape parental resilience. Nonetheless, several limitations should be acknowledged. The studies included in this review displayed heterogeneity in terms of the variability of designs and measurements, limiting the comparability across findings and may constrain generalisability. The predominance of cross-sectional designs also restricts causal inference, making it difficult to determine the directionality between resilience and its associated factors.

### Recommendation for future directions

Future research should prioritise longitudinal study designs to effectively capture the evolution of resilience among parents of children with ASD over time and determine causal relationships. It is also recommended to prioritise evaluating the effectiveness of interventions aimed at enhancing parental resilience. Finally, standardised measurement tools for the parents of children with ASD should be developed to enhance comparability across studies.

## Conclusions

This review highlights that resilience among parents of children with ASD is influenced by the interaction of psychological, social, and contextual factors. Positive psychological factors, together with supportive relationships, favourable socioeconomic conditions, and child characteristics, collectively affect parents’ ability to care for their children. These results emphasise the need for multi-level interventions that enhance both internal coping resources and external supports. Longitudinal research is needed to better understand causal pathways and inform targeted policies and programmes that promote resilience and family well-being.

## Supporting information

S1 Checklist PRISMA 2020 checklist. Checklist used to report compliance with PRISMA 2020 reporting guidelines.
(DOCX)

S2 Checklist PRISMA 2020 for abstract checklist. Checklist used to report compliance with PRISMA 2020 abstract reporting guidelines.
(DOCX)

S1 Table Full search strategy for all databases. Detailed search strings and strategies used across all databases.
(DOCX)

S2 Table Study-level extracted data from included studies. This file contains minimal dataset underlying the findings of this systematic review, including extracted study characteristics and outcome data from all included studies.
(XLSX)
