# Supplementary material for: Factors associated with resilience among parents of children with autism spectrum disorder: A systematic review
Source: PLoS One. 2026 Jun 22;21(6):e0351969. doi: 10.1371/journal.pone.0351969 (PMC13286168; doi:10.1371/journal.pone.0351969)
Supplement: S1 Table — Detailed search strings and strategies used across all databases. (DOCX) [file pone.0351969.s003.docx]

**S1 Table. Full search strategy for all databases.**

| **Database** | **Date searched** | **Filters/ Limits** | **Search string** |
| --- | --- | --- | --- |
| **Web of science** | 2 May 2025 | Publication years: 2019-2025  Document type: Article | TS=(("parent*" OR "mother*" OR "father*") AND ("autism spectrum disorder*" OR "autistic" OR "autism" OR "autistic disorder" OR "neurodevelopmental disorder*" OR "asperger’s" OR "high-functioning autism") AND ("factor*" OR "associate*" OR "related factor*" OR "contributing factor*" OR "determinant*" OR "influencing factor*" OR "predictor*" OR "contributor*" OR "risk factor*" OR "protective factor*") AND ("resilien*" OR "adaptability" OR "adaptation" OR "perseverance" OR "toughness" OR "emotional strength" OR "hardiness" OR "psychological resilience" OR "emotional resilience" OR "psychological adaptation" OR "psychological adjustment" OR "stress resistence" OR "psychological strength")) |
| **Scopus** | 2 May 2025 | Publication years: 2019-2025  No additional filters applied | TITLE-ABS-KEY (("parent*" OR "mother*" OR "father*") AND ("autism spectrum disorder*" OR "autistic" OR "autism" OR "autistic disorder" OR "neurodevelopmental disorder*" OR "asperger’s" OR "high-functioning autism") AND ("factor*" OR "associate*" OR "related factor*" OR "contributing factor*" OR "determinant*" OR "influencing factor*" OR "predictor*" OR "contributor*" OR "risk factor*" OR "protective factor*") AND ("resilien*" OR "adaptability" OR "adaptation" OR "perseverance" OR "toughness" OR "emotional strength" OR "hardiness" OR "psychological resilience" OR "emotional resilience" OR "psychological adaptation" OR "psychological adjustment" OR "stress resistence" OR "psychological strength")) |
| **PubMed** | 2 May 2025 | Publication years: 2019-2025  No additional filters applied | (("parent*" OR "mother*" OR "father*") AND ("autism spectrum disorder*" OR "autistic" OR "autism" OR "autistic disorder" OR "neurodevelopmental disorder*" OR "asperger’s" OR "high-functioning autism") AND ("factor*" OR "associate*" OR "related factor*" OR "contributing factor*" OR "determinant*" OR "influencing factor*" OR "predictor*" OR "contributor*" OR "risk factor*" OR "protective factor*") AND ("resilien*" OR "adaptability" OR "adaptation" OR "perseverance" OR "toughness" OR "emotional strength" OR "hardiness" OR "psychological resilience" OR "emotional resilience" OR "psychological adaptation" OR "psychological adjustment" OR "stress resistence" OR "psychological strength")) |
| **EBSCOhost** | 2 May 2025 | Publication years: 2019-2025  Peer-reviewed journals | AB (("parent*" OR "mother*" OR "father*") AND ("autism spectrum disorder*" OR "autistic" OR "autism" OR "autistic disorder" OR "neurodevelopmental disorder*" OR "asperger’s" OR "high-functioning autism") AND ("factor*" OR "associate*" OR "related factor*" OR "contributing factor*" OR "determinant*" OR "influencing factor*" OR "predictor*" OR "contributor*" OR "risk factor*" OR "protective factor*") AND ("resilien*" OR "adaptability" OR "adaptation" OR "perseverance" OR "toughness" OR "emotional strength" OR "hardiness" OR "psychological resilience" OR "emotional resilience" OR "psychological adaptation" OR "psychological adjustment" OR "stress resistence" OR "psychological strength")) |
